# Bibliometric analysis of research trends in relationship between sarcopenia and surgery

**DOI:** 10.3389/fsurg.2022.1056732

**Published:** 2023-01-06

**Authors:** Tao Liu, Fengjing Song, Deqiang Su, Xiaofeng Tian

**Affiliations:** Department of Hepatopancreatobiliary Surgery, China-Japan Union Hospital of Jilin University, Changchun, China

**Keywords:** sarcopenia, surgery, orthopedics, oncology, bibliometric analysis, visualized study

## Abstract

**Background:**

The relationship between sarcopenia and surgery has attracted an increasing number of researchers in recent years. Our study aimed to identify the current research hotspot and status in this field by using bibliometric and visualization analysis.

**Methods:**

Publications about the relationship between sarcopenia and surgery that met the inclusion criteria were collected from the Science Citation Index Expanded. The bibliometric and visualized studies were performed using VOSviewer, and R.

**Results:**

A total of 2,261 documents on the relationship between sarcopenia and surgery were included in our study. These articles were written by 13,757 authors from 2,703 institutions in 70 countries and were published in 772 journals. The USA is the most productive and influential country in this field (524 publications and 15,220 citations). The Udice French Research Universities was the most productive institution in this field (57 publications), and the University of Alberta had the largest number of citations. Annuals of Surgical Oncology published the most studies in this field. Shen Xian was the most productive author in this field (number of publications = 19), and Baracos Vickie was the most influential author, whose studies in this field had been cited 2,209 times. The cluster analysis was performed and visualized, and the keywords were classified into 6 clusters: Cluster 1 (body composition and nutrition), Cluster 2 (sarcopenia), Cluster 3 (malnutrition and cachexia), Cluster 4 (cancer surgery), Cluster 5 (elderly and frailty), Cluster 6 (neuromuscular scoliosis).

**Conclusion:**

The relationship between sarcopenia and surgery was still a controversial and well-discussed topic in recent years. Our study showed that the study in this field mainly focused on sarcopenia, oncology surgery, orthopedics, and nutrition.

## Introduction

Sarcopenia, as one of the most common geriatric syndromes, was first named by Rosenberg in 1,989 (1), and the concept of sarcopenia was first identified and recognized clearly by EWGSOP (European working group on sarcopenia in older people) in 2010 (2). Sarcopenia is a group of syndromes characterized by progressive loss of skeletal muscle mass, limb dysfunction, and increased risk of adverse events (3). In recent years, with the coming of the aging society, the prevalence of sarcopenia was increasing to 29% in older adults, and for those aged more than 80 years, the number was about 50% (4). Patients with sarcopenia may face an increased risk of falls, fractures, rehospitalization, and even death, due to the decrease in muscle mass and function (5). Many studies have found that many factors may contribute to the development of sarcopenia, such as nutrition status (6), changes in the types of hormones (7), chronic inflammation (8), and mitochondrial dysfunction (9).

The damage and complications caused by surgical procedures have attracted a mass of attention from surgeons in recent years, and sarcopenia has been proven to be a significant factor in rehabilitation for patients who have undergone surgery (10). Moreover, some studies also reported that the surgical procedure may also cause and promote the development of sarcopenia, due to the long stay in bed, malnutrition, and oxidative stress status caused by surgery (11). However, the relationships between sarcopenia and surgery and the underlying mechanism between them were still not fully understood. Figure out the role of sarcopenia in the surgical procedure may improve the quality of life and accelerate the rehabilitation of patients after surgery.

In the past several years, an increasing number of studies focusing on the relationship between sarcopenia and surgical procedure have been published (12, 13). The rapidly increasing publications may confuse the researchers who are interested in the relationship between sarcopenia and surgery and hinder them to trace the frontiers in this field. Bibliometric analysis as a discipline of literature and information science may capture the characteristics of publications and evaluate the trends of studies by qualitative and quantitative methods (14). Bibliometrics performs clustering and other operations through software, and after several times of data analysis, it may help judge the research topic based on the generated clusters and organize the multiple amounts of information systematically (15). However, to the best of our knowledge, few bibliometric analyses were performed in this field. In this study, we aim to provide researchers with the current structures and development of studies focusing on the relationship between sarcopenia and surgery using bibliometric and visualized analysis. The studies in the field of the relationship between sarcopenia and surgery published from 1998 to 2022 were collected and summarized, and the mapping knowledge structure and the research trends were analyzed and visualized.

## Materials and methods

### Data source

Web of Science (WoS, Clarivate Analytics, Philadelphia, PA, USA) is one of the most comprehensive scientific databases and is widely used in many bibliometric studies (16). The Web of Science Core Collection (WoSCC) contains the world's leading scholarly journals, books, and conference proceedings in the sciences, social sciences, arts, and humanities, as well as provides a complete citation network (15, 17). The data included in our study were extracted from the Science Citation Index Expanded (SCI-E) database of WoSCC on August 21, 2022. The impact factor (IF) was collected based on the index provided by the 2021 Journal Citation Reports (JCRs).

### Search strategies

The search terms for sarcopenia were: sarcopenia reduced muscle mass and muscular atrophy. The terms for surgical procedures were: surgery and surgical procedures. The search formula was shown as follows: #1: ((TS = (sarcopenia)) OR TS = (muscular atrophy)) OR TS = (reduced muscle mass); #2: (TS = (surgery)) OR TS = (surgical procedure); #3: #1 AND #2. The search formula #1 may collect the studies on the topic of sarcopenia and #2 contained the publications in the field of surgery. Search formula #3 could obtain the studies containing the both research topics of sarcopenia and surgery.

### Retrieval strategies

The inclusion criteria in this study were set as below: (a) articles focusing on the relationship between sarcopenia and surgical procedure; (b) document types identified as articles or review articles; (c) studies published from January 1, 1998, to August 21, 2022. The exclusion criteria were set as follows: (a) Meeting abstract; Proceeding paper; Editorial material; Early access; Letter; Book Chapters; Correction; Retracted publication; (b) non-English publications. (c) documents without sufficient information. The studies met the inclusion criteria and the exclusion criteria were included in this study.

### Documents extraction

The full records of the identified documents were extracted from the WoS database. The information contained in records was as below: title, authors, keywords, journals, abstract, year of publication, countries/regions, affiliates, research direction, funding agencies, and H-index. All the records mentioned above were summarized in Microsoft Office Excel 2019.

### Bibliometric and visualized analysis

Microsoft Office Excel 2019 (Microsoft Corporation, Santa Rosa, CA, USA) was used to perform the descriptive analysis. The predicted publication curve based on the number of publications in each year was performed by using the Trend Line function in Microsoft Office Excel 2019. The network maps of collaborative and cited relations of countries, institutes, and authors were performed by using VOSviewer (18) version 1.6.18 (Leiden University Center for Science and Technology Studies, Leiden, the Netherlands). In the maps generated by using VOSviewer, different nodes present different terms, such as authors, countries, and so on. The lines between nodes mean the relationships between terms, and total link strength (TLS) represents the connection strength between the nodes (19). Moreover, an online bibliometric platform (https://bibliometric.com/) and Package Bibliometrix built in R software (v4.0.1, R Foundation, Vienna, Austria) were also used to visualize the collaboration of countries and the cited relation between journals.

## Results

### Overview information and trends of publications

**Figure 1:**
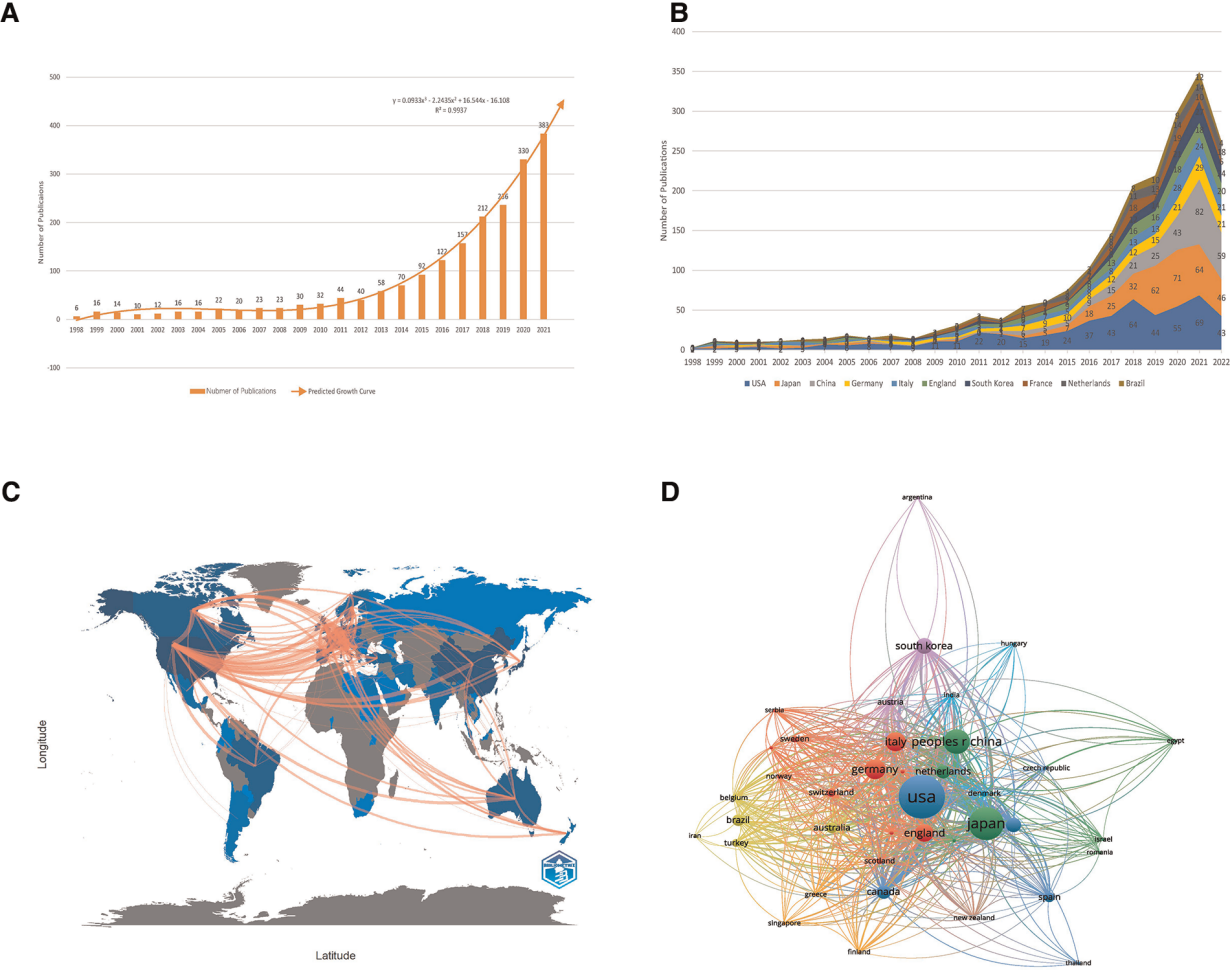
Analysis of general information and countries of publications. (**A**) The number of publications about the relationship between sarcopenia and surgery from 2000 to 2021; (**B**) The number of publications of the top 10 most productive countries from 200 to 2022; (**C**) Map of collaborative relationships between countries; (**D**) Map of citation relationships between countries. Different nodes present different countries. The size of nodes is proportional to the number of publications in each country, and the color of the lines between two nodes presents the relationship that the countries cited each other. The thicker the lines between two nodes, the higher the frequency with that they are cited to each other.

A total of 2,261 documents on the relationship between sarcopenia and surgery were included in our study. These articles were written by 13,757 authors from 2,703 institutions in 70 countries and were published in 772 journals. From 1998 to 2021, the number of publications in this field was growing rapidly. Before 2008, about 20 articles in this field were published, and the number increased several times in the years after 2008. More than 100 studies published since 2016, and the number passed 200 in 2018, and 2 years later, the number of publications had increased to 330 (Figure 1A). The predicted growth equation established by using Microsoft Office Excel 2019 were shown in the Figure 1A: *y* = 0.0933*x*^3 ^− 2.2435*x*^2 ^+ 16.544*x* − 16.108, *R*² = 0.9937. In this equation: the “*x*” presents the year and the “*y*” means the predictive number of publications per year. The predictive numbers of publications in 2022 and in 2030 are 453 and 1,440, respectively.

### Analysis of countries or regions

The number of publications of the top ten most productive countries was summarized in Figure 1B according to their publication year. The USA dominate this field before 2015, and the publication number of Japan and China increased rapidly since 2016. In the 3 years from 2019 to 2021, the USA was no longer the most productive country in this field. The researchers in East Asia, especially in Japan and China, published a large number of publications on the relationship between sarcopenia and surgery, and it might be due to East Asia's severely aging societies (20, 21). The detailed information of the most productive 10 countries was summarized in Table 1. Undoubtedly, the USA was the most influential country in the field of the relationship between sarcopenia and surgery, due to its highest H-index and its most publications and cited times (Table 1).

**Table 1:** The top 10 countries with the largest number of publications on the relationship between sarcopenia and surgery.

	H-Index	Numbers	Citations	Average citations per publication
USA	62	524	15,220	29.05
Japan	36	353	5,057	14.33
China	29	283	2,981	10.53
Germany	31	170	5,407	31.81
Italy	36	163	5,753	35.3
England	37	145	4,986	34.39
South Korea	15	122	1,151	9.43
France	26	107	3,992	37.31
Netherlands	32	99	5,022	50.73
Brazil	18	79	1,062	13.44

Collaboration relationships between countries were visualized in Figure 1C. In this map, the color shape represents the number of publications in each country, and the line represents the collaboration between countries. The USA, Canada, and European countries collaborated most frequently in the past 20 years. The citation relationship between countries were shown in Figure 1D. The size of nodes is proportional to the number of publications in each country, and the color of the lines between two nodes presents the relationship that the countries cited each other. The thicker the lines between two nodes, the higher the frequency that they are cited to each other. The USA, Germany, China, and Japan had the most frequent citation relation (Figure 1D).

### Analysis of institutions

**Figure 2:**
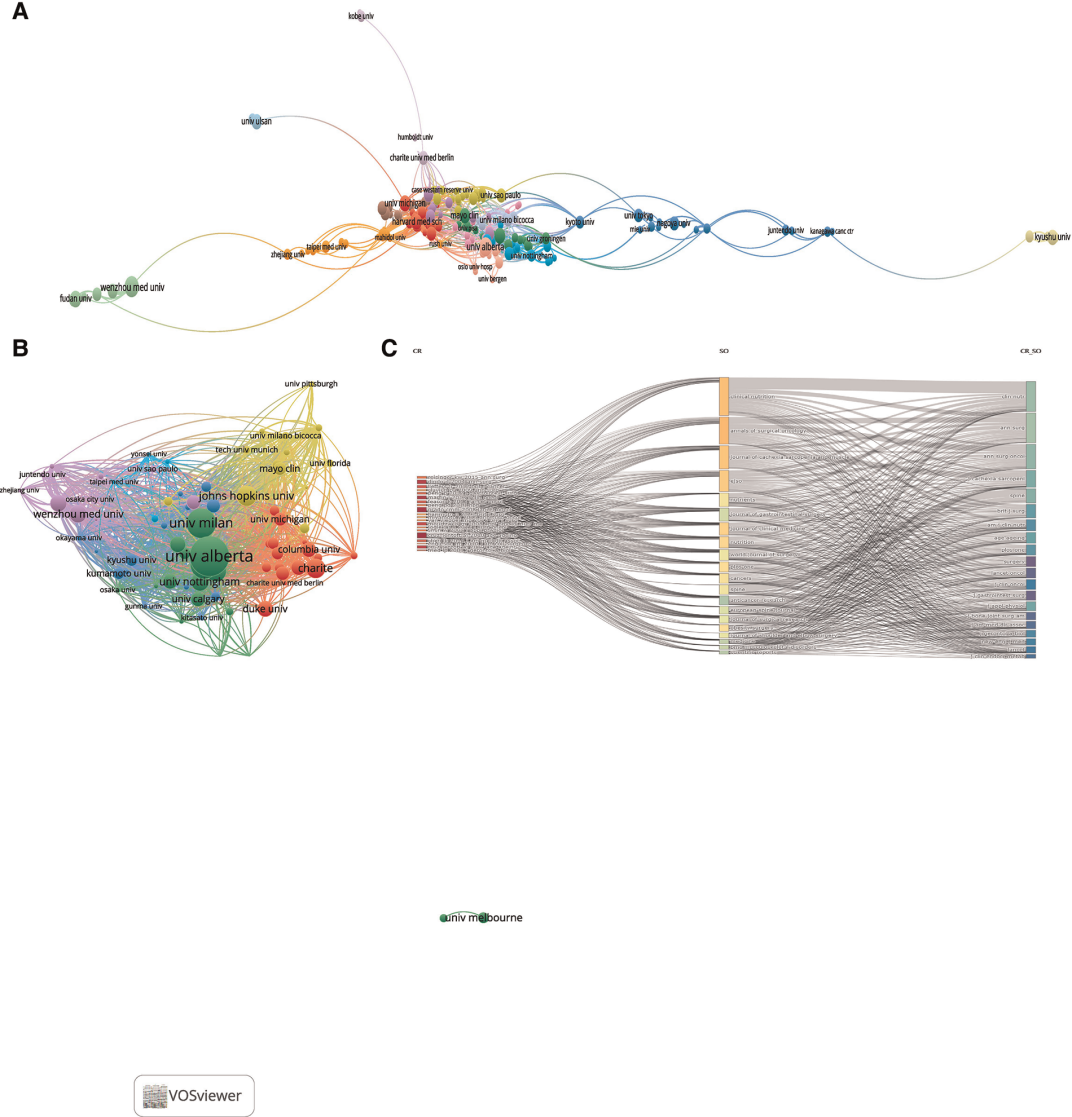
Analysis of relationships between institutions and the cited relationship between journals. (**A**) Map of collaborative relationships between institutions: different nodes present different institutions. The size of nodes is proportional to the number of publications in each institution, and the color of the lines between two nodes presents the collaborative relationship between institutions; (**B**) Map of citation relationships between institutions: the size of nodes represents the number of citations and the line means their citation relationship; (**C**) Map of cited relationships between journals: the left column means the publications with the most co-cited times, the middle column means the journals with the most co-cited times, and the right column means the journals with the most citations.

A total of 2,703 institutions conducted studies on the relationship between sarcopenia and surgery. The top 10 institutions with the largest number of publications were summarized in Table 2. The Udice French Research Universities was the most productive institution in this field (number of publications = 57), and Harvard University had the highest H-index (H-index = 23). The collaborative relationships between institutions were visualized in Figure 2A. Harvard University, the University of Alberta, and the University Of Milano-Bicocca had the highest TLS, and they were at the center of the collaboration map. Figure 2B showed the citation relationships between institutions. The size of nodes represents the number of citations and the line means their citation relationship. The University of Alberta received the most citations (cited times = 3,277).

**Table 2:** The top 10 institutions with the largest number of publications on the relationship between sarcopenia and surgery.

Institutions	H-Index	Citations	Numbers	Average citations per publication
Udice French Research Universities	19	1,523	57	26.72
Harvard University	23	1,565	52	30.1
Wenzhou Medical University	14	937	41	22.85
Institut national de la santé et de la recherche médicale	16	1,290	39	33.08
University of California System	20	1,503	39	38.54
Assistance Publique Hopitaux Paris	17	1,428	37	38.59
University of London	14	1,039	35	29.69
University of Alberta	17	3,277	34	96.38
Free University of Berlin	12	1,233	30	41.1
University of Michigan System	16	977	28	34.89
University of Texas System	13	847	28	30.25

### Analysis of journals

A total of 722 journals published studies on the relationship between sarcopenia and surgery. The top 10 journals that published the largest number of studies in this field were summarized and ranked in Table 3. Three journals published more than 40 studies: Annuals of Surgical Oncology (number of publications = 46), Clinical Nutrition (number of publications = 45), and Journal of Cachexia Sarcopenia and Muscle (number of publications = 41). Clinical Nutrition had been cited the most times (3,774 cited times), and the Journal of Cachexia Sarcopenia and Muscle had the highest impact factors (IF = 12.063). The cited relationships in journals could be found in Figure 2C. Clinical Nutrition, Annals of Surgery, and Annuals of Surgical Oncology were the most cited journals in this field.

**Table 3:** The journals with more than 20 publications on the relationship between sarcopenia and surgery.

Journals	Citations	Numbers	H-Index	Impact factors
Annuals of Surgical Oncology	1518.00	46	21	4.339
Clinical Nutrition	3774.00	45	19	7.643
Journal of Cachexia Sarcopenia and Muscle	990.00	41	17	12.063
EJSO	849.00	37	14	4.037
Journal of Clinical Medicine	114.00	29	7	4.964
Nutrition	376.00	26	10	4.893
Plus One	414.00	25	14	3.752
Obesity Surgery	415.00	24	13	3.479
Cancers	117.00	22	7	6.575
Spine	681.00	22	14	3.241
Nutrients	133.00	22	7	6.706
World Journal of Surgery	380.00	22	11	3.282
Journal of Shoulder and Elbow Surgery	524.00	21	11	3.507
Journal of Surgical Research	271.00	20	10	2.417

### Analysis of authors

**Figure 3:**
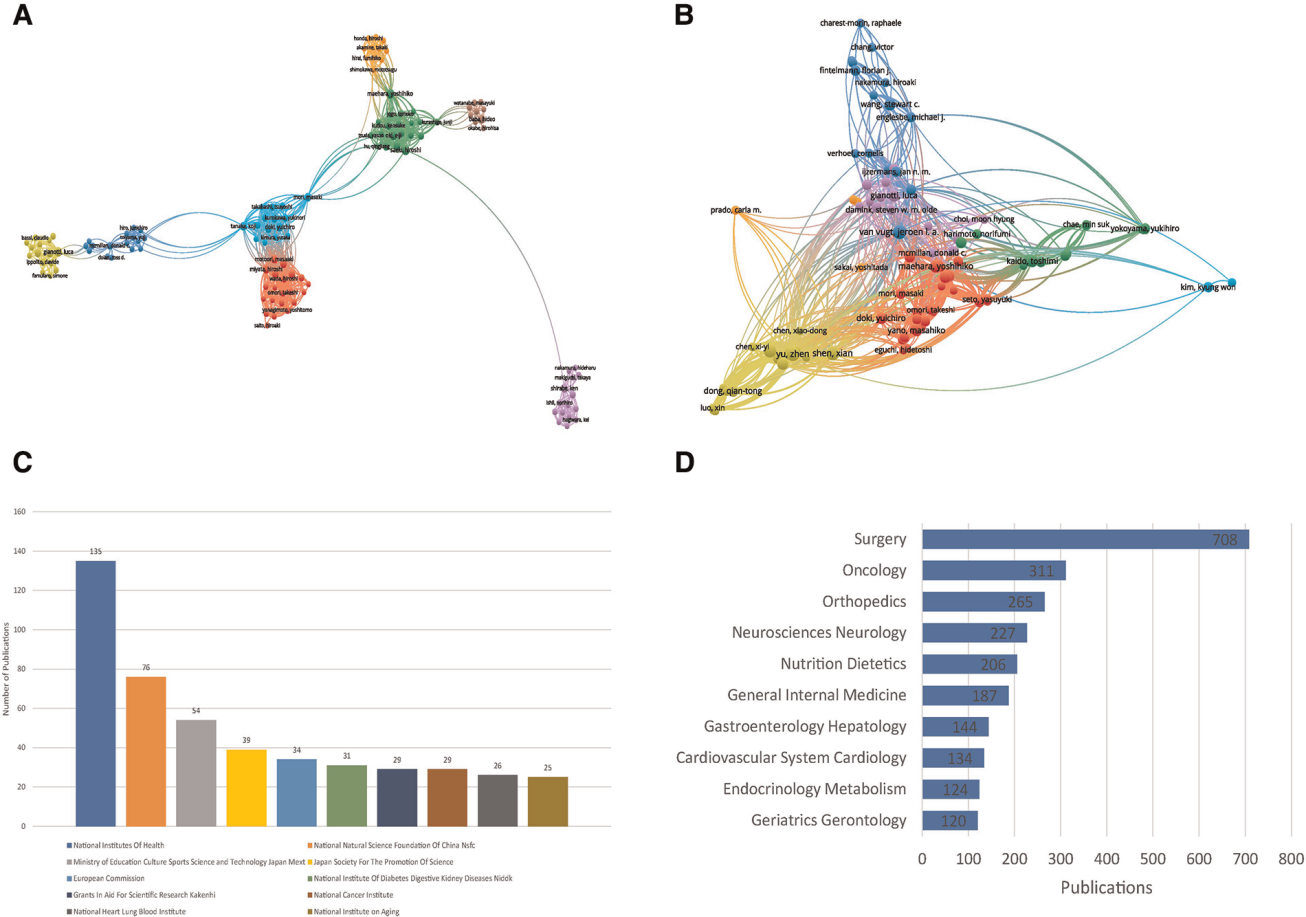
Information of authors, funding agencies, and research directions: (**A**) Map of collaborative relationships between authors: the size of nodes represents the number of publications and the line means their collaborative relationship; (**B**) Map of cited relationships between authors: the size of nodes represents the number of citations and the line means their citation relationship; (**C**) Distribution of the top 10 funding agencies; (**D**) The information of top 10 research directions.

The detailed information including citation times, number of publications, and H-index of the most productive and influential authors in the field of the relationship between sarcopenia and surgery were shown in Tables 4, 5 respectively. Shen Xian was the most productive author in this field (number of publications = 19), and Baracos Vickie was the most influential author, whose studies in this field had been cited 2,209 times. The collaboration relationships of authors were visualized in Figure 3A. The map of author relationships was divided into 8 clusters and the relationships were few and far between clusters. Figure 3B showed the citation relationships between authors, and the cited relationships were wide (Figure 3B).

**Table 4:** The top 10 most productive authors focusing on the relationship between sarcopenia and surgery.

Authors	Citations	Average citations per publication	Numbers	H-Index
Shen Xian	805	42.37	19	12
Yu Zhen	793	44.06	18	12
Van Vugy Jeroen	996	55.33	18	12
Zhuang Chengle	834	49.06	17	12
Chen Xiaolei	207	13.8	15	7
Baracos Vickie	2,209	147.27	15	10
Wang Stewart	511	36.5	14	10
Gianotti Luca	349	26.85	13	8
Huang Dongdong	665	51.15	13	8
Maehara Yoshihiko	411	34.25	12	10

**Table 5:** The top 10 most influential authors focusing on the relationship between sarcopenia and surgery.

Authors	Citations	Average citations per publication	Numbers	H-Index
Baracos Vickie	2,209	147.27	15	10
Pawlik Timothy	1,164	129.33	9	9
Van Vugy Jeroen	996	55.33	18	12
Zhuang Chengle	834	49.06	17	12
Shen Xian	805	42.37	19	12
Yu Zhen	793	44.06	18	12
Wang Sulin	696	69.6	10	8
Wang Stewart	511	36.5	14	10
Lou Neng	500	353	6	5
Maehara Yoshihiko	411	34.25	12	10

### Analysis of funding agencies and research directions

The top 10 funding agencies were summarized in Figure 3C. The National Institutes of Health was the funding agency that funded the largest number of studies (number of funded studies = 135). The National Natural Science Foundation of China and the Ministry of Education Culture Sports Science and Technology Japan Mext funded 76 and 54 studies respectively. Of the top 10 funding agencies, half of them were from the USA. The research directions were summarized in Figure 3D. Most of the studies in this field were focusing on surgery, oncology, and orthopedics.

### Analysis of influential studies

**Figure 4:**
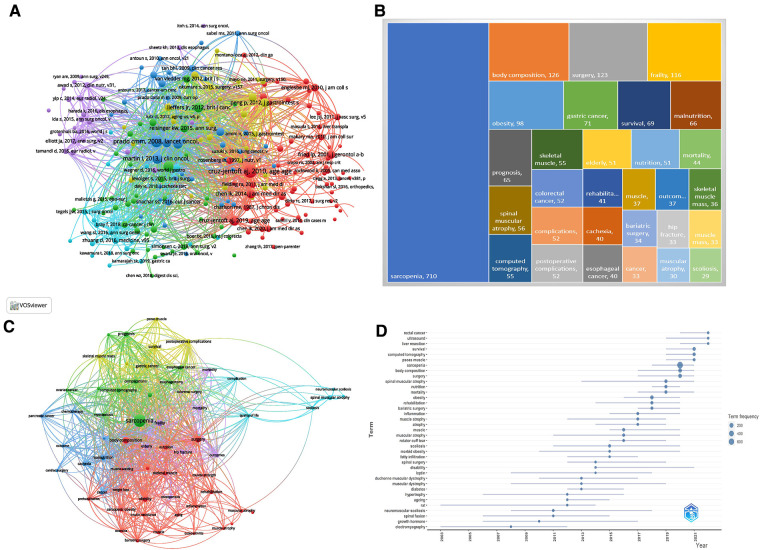
Information of the co-cited publications and keywords: (**A**) Map of the co-cited relationships of publications: the size of nodes represents the number of co-cited times and the line means their co-cited relationship; (**B**) Distribution of the top 30 keywords; (**C**) The map of clusters of keywords: the size of nodes represents the number of co-occurrence times and the line means their co-occurrence relationship. Different color means different clusters; (**D**) The map of keywords and their occurrence year.

The top 10 papers with the largest cited times were shown in Table 6. All of them were published between 2000 and 2017 and were cited more than 300 times. An article entitled “ESPEN guidelines on nutrition in cancer patients” published in Clinical Nutrition has been cited 1,248 times, and was the publication with the most cited times. Figure 4A showed the co-cited relationships in this field. Co-cited publications could reflect the research foundation in this field. A guideline entitled “Sarcopenia: European consensus on definition and diagnosis: Report of the European Working Group on Sarcopenia in Older People” had received the most co-cited times (TLS = 4,444).

**Table 6:** The top 10 studies with the most citations on the relationship between sarcopenia and surgery.

Title	Journal	Author	Publication year	Citation
ESPEN guidelines on nutrition in cancer patients	Clinical Nutrition	Arends, J	2017	1,248
Prognostic impact of disease-related malnutrition	Clinical Nutrition	Norman, K	2008	857
The results of repair of massive tears of the rotator cuff	Journal of Bone and Joint Surgery	Gerber, C	2000	817
Sarcopenia is associated with postoperative infection and delayed recovery from colorectal cancer resection surgery	British Journal of Cancer	Lieffers, JR	2012	502
ESPEN expert group recommendations for action against cancer-related malnutrition	Clinical Nutrition	Arends, J	2017	453
Effect of Caloric Restriction or Aerobic Exercise Training on Peak Oxygen Consumption and Quality of Life in Obese Older Patients With Heart Failure With Preserved Ejection Fraction A Randomized Clinical Trial	JAMA	Kitzman, DW	2016	397
Rapidly Progressive Diaphragmatic Weakness and Injury during Mechanical Ventilation in Humans	American Journal of Respiratory and Critical Care Medcine	Jaber, S	2011	390
Impact of Sarcopenia on Outcomes Following Resection of Pancreatic Adenocarcinoma	Journal of Gastrointestinal Surgery	Peng, P	2012	355
Assessment and management of nutrition in older people and its importance to health	Clinical Interventions in Aging	Ahmed, T	2010	321
Functional Compromise Reflected by Sarcopenia, Frailty, and Nutritional Depletion Predicts Adverse Postoperative Outcome After Colorectal Cancer Surgery	Annals of Surgery	Reisinger, KW	2015	312

### Analysis of keywords

Author keywords were extracted from all the papers included in our study. The top 30 most frequent keywords were summarized in Figure 4B. “Sarcopenia”, “body composition”, and “surgery” were the top three keywords with the most occurrences. The cluster analysis was performed and shown in Figure 4C. Keywords were classified into 6 clusters: Cluster 1 (body composition and nutrition), Cluster 2 (sarcopenia), Cluster 3 (malnutrition and cachexia), Cluster 4 (cancer surgery), Cluster 5 (elderly and frailty), Cluster 6 (neuromuscular scoliosis). Figure 4D showed the development of keywords over the past 20 years. At the beginning of this century, researchers in this field paid more attention to neuromuscular scoliosis, and gradually, the emphasis was changed to muscle atrophy. After 2016, sarcopenia had become the most significant research point in this field.

## Discussion

In our study, the SCI-E database was used to obtain information on publications on the relationship between sarcopenia and surgery due to its authoritative and comprehensive academic collection (22). The search results of publications on the relationship between sarcopenia and surgery in the SCI-E database may represent the research status in this field comprehensively. Moreover, our study followed the procedures of bibliometric studies suggested previously: (a) identification of search strategies; (b) identification of period and database; (c) identification of inclusion and exclusion criteria; (d) data transformation; (e) data analysis (23).

The relationship between sarcopenia and surgery was still a concern and discussed topic in recent years due to the significant roles played by sarcopenia in surgical procedures, as well as the prognosis after surgeries (24, 25). A study that enrolled 480 older patients with gastrointestinal and hepatobiliary pancreatic tumors showed that patients with poor muscle strength may have poor outcomes after their first resection surgeries (26). Similarly, a study conducted in South Korea showed a similar conclusion: among the patients with resectable pancreatic ductal adenocarcinoma, those with sarcopenia may have lower overall survival and recurrence-free survival after surgery than the patients without sarcopenia (27). Despite the surgeries mentioned above, many studies also suggested the prognosis value of sarcopenia in minimally invasive distal gastrectomy (28), cardiovascular surgery (29), pheochromocytoma and paraganglioma surgery (30), percutaneous kyphoplasty (31), and so on. Otherwise, many studies suggested that the surgery may cause the occurrence and aggravation of sarcopenia (32). A multi-center study conducted in the USA showed that sarcopenia is more prevalent in patients with inflammatory bowel disease undergoing surgery, while the clinical nutrition markers including the body mass index and albumin were similar in the population (33). It is also reported that patients who underwent different methods of pancreatoduodenectomy may also have different changes in skeletal muscle cross-sectional area and visceral fat area, and the incidence of sarcopenia in both surgical procedures continued to increase (34).

Many studies paid more attention to the role of nutrition in sarcopenia and surgery (35–37). A prospective study from China showed that the combined systemic inflammatory immunity index and prognostic nutritional index scores as an indicator of nutrition status may predict the incidence of sarcopenia for patients with advanced gastric cancer who underwent radical surgical resection (38). Moreover, a cohort study conducted in Japan reported that the protein intake may affect the skeletal muscle mass of patients who underwent kidney transplantation, and suggested that a protein intake of more than 0.72 g/kg IBW/day may provide the patients with improved skeletal muscle mass (37). A study based on the data from National Health and Nutrition Examination Survey also suggested the inverse relationship between serum iron and muscle mass (39).

For the analysis of journals, in the top 10 journals with the most publications in this field, 3 journals focused on the field of cancer, and the same number of journals majored in nutrition, which suggested that the relationship between sarcopenia and oncological surgery and the role of nutrition in this field were attracted the most attention. The results of the journal analysis were consistent with the research trend in this field. In the overall bibliometric results, our study had shown that the USA played the most influential role in this field. The USA has published the largest number of publications on the relationship between sarcopenia and surgery and has the highest H-index and the most citations. Similarly, in the analysis of institutions, four of the most productive institutions were from the USA.

Co-cited studies may show the fundamental studies in this field. As shown in Figure 4A, a practice guideline based on The European Working Group on Sarcopenia in Older People (EWGSOP) was the publication with the most co-cited times. This paper was the first consensus on sarcopenia and stated the definition and diagnosis of sarcopenia for the first time (40). However, this consensus was revised in 2019 (13), and the updated guideline reported new insights into sarcopenia: more attention to muscle strength, a more precise clinical algorithm, and more clear cut-off points. The second fundamental study in this field was a population study focusing on the prevalence and clinical implications of sarcopenic obesity in patients with solid tumors of the respiratory and gastrointestinal tracts (41). Similarly, the co-cited study with the third largest number of citations reported that skeletal muscle depletion may predict poor outcomes for patients with cancer cachexia (42). Consistent with the result we mentioned above, more research topics on the relation between sarcopenia and surgery focused on cancer.

Six clusters were generated from the co-occurrence analysis of keywords, and these clusters may indicate the research topics on the relationship between sarcopenia and surgical procedures. Just as we mentioned above, body composition and nutrition are one of the most attractive topics in this field. Body composition is not only the indicator of sarcopenia but is also identified as one of the most significant measurements for the diagnosis of sarcopenia (43). Cluster 3 focused on malnutrition and cachexia. Malnutrition, cachexia, and sarcopenia were similar body statuses with metabolism disorders and were always discussed together (44). Elderly and frailty as the main topic of cluster 4 were also hot research points in recent years due to the understanding of sarcopenia in orthopedics, especially the frailty fractures in older adults (45). Older individuals with poor muscle status may not only face a high incidence of fractures and falls but also poor survival and walking ability after surgeries due to fractures (46, 47). Cluster 6 was the neuromuscular scoliosis. Neuromuscular scoliosis is an abnormal curvature of the spine due to neuromuscular disease (48), and this topic was studied more in the early time in this field.

There were some limitations in our study. First, sarcopenia as a new concept may have rapid development in recent years, while some articles without a clear conception of sarcopenia may fail to be searched. Next, all the publications in our study were attained from SCI-E, one of the most comprehensive and well-known databases. Therefore, many valuable studies indexed in other databases but not in SCI-E may be ignored in our study. Thirdly, some studies published in the recent year may receive fewer citations than those published earlier. Lastly, all the articles included in this study were published in the English language and may contribute to the loss of publications in other languages.

## Conclusion

This study revealed the research trend and status of the relationship between sarcopenia and surgery in the recent 20 years. The USA is the most productive and influential country in this field (524 publications and 15,220 citations). The Udice French Research Universities was the most productive institution in this field (57 publications), and the University of Alberta had the largest number of citations. Annuals of Surgical Oncology published the most studies in this field. Shen Xian was the most productive author in this field (number of publications = 19), and Baracos Vickie was the most influential author, whose studies in this field had been cited 2,209 times. The cluster analysis was performed and shown in Figure 4C. Keywords were classified into 6 clusters: Cluster 1 (body composition and nutrition), Cluster 2 (sarcopenia), Cluster 3 (malnutrition and cachexia), Cluster 4 (cancer surgery), Cluster 5 (elderly and frailty), Cluster 6 (neuromuscular scoliosis).

## Data Availability

The original contributions presented in the study are included in the article/Supplementary Material, further inquiries can be directed to the corresponding author/s.
